# Social Vulnerability and Biological Aging in New York City: An Electronic Health Records-Based Study

**DOI:** 10.1007/s11524-024-00948-7

**Published:** 2025-01-14

**Authors:** Pablo Knobel, Elena Colicino, Itai Kloog, Rachel Litke, Kevin Lane, Alex Federman, Charles Mobbs, Maayan Yitshak Sade

**Affiliations:** 1 Department of Environmental Medicine and Climate Science, Icahn School of Medicine at Mount Sinai, 1 Gustave L. Levy Place, Box 1057, New York, NY 10029 USA; 2 Nash Family Department of Neuroscience, Icahn School of Medicine at Mount Sinai, Friedman Brain Institute, New York, NY USA; 3 Department of Environmental Health, Boston University School of Public Health, Boston, MA USA; 4 Division of General Internal Medicine, Icahn School of Medicine, New York, NY USA

**Keywords:** Social vulnerability, Biological aging, Disparities, Electronic health records, New York City

## Abstract

Chronological age is not an accurate predictor of morbidity and mortality risk, as individuals’ aging processes are diverse. Phenotypic age acceleration (PhenoAgeAccel) is a validated biological age measure incorporating chronological age and biomarkers from blood samples commonly used in clinical practice that can better reflect aging-related morbidity and mortality risk. The heterogeneity of age-related decline is not random, as environmental exposures can promote or impede healthy aging. Social Vulnerability Index (SVI) is a composite index accounting for different facets of the social, economic, and demographic environment grouped into four themes: socioeconomic status, household composition and disability, minority status and language, and housing and transportation. We aim to assess the concurrent and combined associations of the four SVI themes on PhenoAgeAccel and the differential effects on disadvantaged groups. We use electronic health records data from 31,913 patients from the Mount Sinai Health System (116,952 person-years) and calculate PhenoAge for years with available laboratory results (2011–2022). PhenoAge is calculated as a weighted linear combination of lab results, and PhenoAgeAccel is the differential between PhenoAge and chronological age. A decile increase in the mixture of SVI dimensions was associated with an increase of 0.23 years (95% CI 0.21, 0.25) in PhenoAgeAccel. The socioeconomic status dimension was the main driver of the association, accounting for 61% of the weight. Interaction models revealed a more substantial detrimental association for women and racial and ethnic minorities with differences in leading SVI themes. These findings suggest that neighborhood-level social vulnerability increases the biological age of its residents, increasing morbidity and mortality risks. Socioeconomic status has the larger detrimental role among the different facets of social environment.

**Supplementary Information:**

The online version contains supplementary material available at 10.1007/s11524-024-00948-7.

## Introduction

The proportion of individuals ≥ 65 years old is projected to grow from 1:11 in 2022 to 1:6 of the global population by the year 2050: an increase from 771 million to 1.6 billion [1]. Biologically, aging results from the buildup of diverse molecular and cellular damage that leads to declining physical and mental capacities. Over time, this decline translates into increased morbidity and mortality risk [2]. Thus, higher life expectancy can result in years spent in ill health instead of healthy aging [3]. However, chronological age is not a perfect measure of risk, as individuals’ aging processes are diverse [4]: some individuals become frail and dependent, while others remain entirely autonomous. Aging phenotypes are diverse, but the underlying mechanisms are common. The geroscience hypothesis points toward seven main mechanisms, including changes in metabolism, inflammation, and epigenetics [5], which can be measured through biomarkers. However, a single biomarker is unlikely to capture the complexity of aging processes. By incorporating diverse biomarkers linked to these underlying aging mechanisms, biological age metrics can reflect aging-related morbidity and mortality risk better than chronological age [6]. Phenotypic age (PhenoAge) is a validated biological age measure incorporating chronological age and biomarkers from blood samples commonly used in clinical practice. Phenotypic age acceleration (PhenoAgeAccel) measures the degree to which a person is younger or older than their chronological age [7] and predicts the risk of morbidity and mortality better than chronological age [7, 8]. The use of common clinical biomarkers facilitates the use of PhenoAge in large retrospective cohorts using EHR. Contrarily, histology-based biomarkers, as DNA methylation, are dependent on specific tests not routinely done in clinical settings [9].

The heterogeneity of age-related decline is not random, as personal and environmental exposures can promote or impede healthy aging. The Social Hallmarks of Aging highlighted how personal-level social stressors are key determinants of aging-related health [10]. For example, women, despite generally living longer than men, tend to be frailer and perform worse in physical function examinations [11], and non-Hispanic Blacks and Hispanics have accelerated biological aging measures through a different biomarker-based biological age [12]. Originally, the Social Hallmarks of Aging left out the broader socioeconomic context. However, the neighborhood someone lives in has been linked to many aging-related outcomes [13]. For example, the socioeconomic, racial, and ethnic composition of the neighborhood of residence has been associated with increased odds of frailty [14], and negative neighborhood perception is associated with reduced physical activity and increased sedentary time [15]. Safety is crucial for seniors’ well-being [16], with associations between self-reported safety and improved self-reported health [17]. Both physical and socioeconomic neighborhood characteristics have been linked to biological aging measures through epigenetic metrics, including increases in epigenetic ages associated with neighborhood deprivation [18].

The Center for Disease Control and Prevention (CDC) developed the Social Vulnerability Index (SVI) as a composite index to account for the socioeconomic environment [19]. The SVI encompasses social factors organized into four themes: socioeconomic status, household composition and disability, minority status and language, and housing and transportation. The four themes of the index represent different facets of the social environment that are always present simultaneously. However, social environment facets are usually studied independently. These independent analyses raise questions about the combined effects of social exposures and their relative contributions. Concurrent time and space-varying exposures might influence each other’s impact on aging. Therefore, only considering a single exposure may underestimate or overestimate its association, without accounting for potential effect addition, amplification, or confounding. In other words, it is critical to tackle social environment exposures from a social exposome perspective—as concurrent omnipresent exposures that concurrently and systematically impact human health and development [20].

Social environment exposures are unequally distributed across the USA, creating an intersection between personal and neighborhood-level social stressors, with vulnerable populations often being exposed to harsher conditions. For example, in more segregated areas, Black and Hispanic residents tend to live near schools with lower proficiency rates and higher numbers of students on free or reduced-price lunch compared to Whites. They also face significantly more neighborhood violent crime than Whites and those in less segregated areas [21]. Disadvantaged groups thus suffer from the combination of harmful neighborhood-level exposures and personal-level stressors. These neighborhood-level exposures can influence individual behaviors, and individual characteristics can change how neighborhoods are perceived and interacted with [22], potentially leading to larger exposure and aging disparities [23]. The intersection of social stressors is layered on top of the “double jeopardy”—or how disadvantaged populations face more frequent and intense exposure to environmental stressors and are less equipped to manage these risks due to limited awareness and marginalization from the political process [24].

In this study, we assess the concurrent and combined associations of the four themes of SVI on PhenoAgeAccel using an exposure-mixture approach. We leverage 11 years of electronic health records (EHR) data from the Mount Sinai Health System (MSHS). Additionally, we evaluate the differential effects by sex as well as race and ethnicity.

## Methods

### Study Population

This retrospective cohort study includes New York City residents who were 65 years or older and have been treated at the MSHS between 2011 and 2022. Data was obtained through the Mount Sinai Data Warehouse. We excluded individuals without valid information on race/ethnicity (8.1%) or insurance (4.0%), as well as person-years without address (1.4%) or recorded measures of the biomarkers needed to calculate PhenoAgeAccel (65.8%)—mostly made by person-year where the participants did not have any visits. This study was approved by the Institutional Review Board of Mount Sinai (STUDY 22–01400), and a waiver of informed consent was granted.

### Exposure Assessment

The SVI encompasses social factors (details on each theme can be found in Table S1) extracted from the US Census and American Community Survey calculated for each non-zero population census tract, an administrative boundary which aims to be demographically homogeneous and has around 4000 inhabitants [25]. All the variables are weighted equally, as are the four themes. SVI and each theme range from zero to one, with higher values indicating greater vulnerability [26]. Addresses in the MSHS are updated in every patient encounter in which the patient reports a change of address. We linked SVI to participants annually based on the last reported address on file. We calculated SVI using *findSVI* [27] and *tidycensus* [28] in R [29].

### PhenoAgeAccel Calculation

We derived PhenoAge for each person-year from laboratory results using the formula described by Levine et al. [30]. PhenoAge is calculated as a weighted linear combination of lab results transformed into units of years using two parametric proportional hazard models [30]. We averaged all the available measures of each required biomarker for each year. Most required biomarkers are routinely drawn: albumin, creatinine, glucose, mean cell volume, alkaline phosphatase, red cell distribution width, and white blood cell count. However, C-reactive protein and lymphocyte percent might be drawn routinely but are often obtained when a concern arises for infections, hematologic, liver, or renal disease. To avoid selection bias by limiting our population to a sicker group that has these two tests performed, we imputed C-reactive protein (90.83%) and lymphocyte percent (24.06%). We used the predictive mean matching (pmm) method from the *mice* [31] package in R because it guarantees imputations within the range of observed data and has been shown to have low root mean square error, fast computation time, and minimal challenges in implementation [32]. We defined PhenoAgeAccel, our outcome of interest, as the differential between chronological age and PhenoAge: a positive value of PhenoAgeAccel means accelerated aging, and a negative value means decelerated aging.

### Statistical Analysis

We used quantile g computation (qGcomp) [33] to assess the combined association of the four SVI themes and assess the relative contribution of each theme to PhenoAgeAccel. This approach is an extension of the g-computation method and estimates the overall association of the mixture with the outcome and outputs a weight with direction for each exposure in the mixture, allowing us to identify the most highly weighted contributors for each association direction. Exposures contributing to the mixture are combined into a supervised weighted index, defined by deciles of the exposures. The weighted contribution of each exposure is estimated relative to the contribution of the other exposure effects. The overall mixture effect is interpreted as the expected change in PhenoAgeAccel associated with increasing all exposures by one decile simultaneously [34]. We adjusted our models for sex (male, female), race and ethnicity combined (American Indian or Alaska Native, Asian, Black or African-American, Hispanic, Native Hawaiian or Pacific Islander, Other, White), and type of insurance (Medicaid, Medicare, Other, Private insurance, Self-pay). To estimate a time-varying baseline risk, we adjusted the model for an indicator variable of year of observation [35]. We assessed the potential interaction of the exposures with sex (male vs female), as well as race and ethnicity (non-Hispanic White vs other racial and ethnic groups). To evaluate the potential effect of imputing C-reactive protein and lymphocytes, we repeated the qGcomp analysis including only person-years without any imputed lab results as sensitivity analysis. Additionally, we used cluster-based bootstrapping to evaluate the potential clustering and autocorrelation among participants’ repeated measures as well as participants living in the same census tracts. We used the *qgcomp* [33] and *qgcompint* [36] packages in R [29]. The estimates are reported for a decile change in all SVI themes.

## Results

We included 116,952 person-years from 31,913 participants. The average participant’s age was 70.21 years. One-third of the participants were male, almost half were white, and a quarter were Hispanic. Variability at baseline in the socioeconomic status and household characteristics is higher than in the other CDC-SVI themes (Table 1). Correlations among SVI themes range from 0.84 in racial and ethnic minority status and socioeconomic status to 0.10 for household characteristics and household type and transportation, the latter having consistently lower correlations than the rest across comparisons (Figure S1). Our stratified analysis by sex and race and ethnicity looked at different subsets of the population. When stratifying the sample by sex, there is a higher percentage of Hispanics and Blacks and a lower percentage of Whites among women compared to men. Age, insurance, and SVI values remain similar. When stratifying the sample by race, there is a higher percentage of men, fewer people covered by Medicaid, and more people covered by Medicare among Whites compared to the other groups. SVI values are consistently lower among Whites (Table S2).

**Table 1:** Descriptive statistics for the study population at baseline

Variable	Overall
*n*	31,913
Sex = male (%)	10,869 (34.1)
Age (mean (SD))	70.21 (8.49)
Race and ethnicity combined (%)	
*American Indian or Alaska Native*	35 (0.1)
*Asian*	1494 (4.7)
*Black or African-American*	5105 (16.0)
*Hispanic*	8241 (25.8)
*Native Hawaiian or Pacific Islander*	17 (0.1)
*Other*	3057 (9.6)
*White*	13,964 (43.8)
Insurance type (%)	
*Medicaid*	1124 (3.5)
*Medicare*	22,626 (70.9)
*Other*	1435 (4.5)
*Private insurance*	6418 (20.1)
*Self-pay*	310 (1.0)
Overall SVI (mean (SD))	0.58 (0.28)
Socioeconomic status (mean (SD))	0.50 (0.31)
Household characteristics (mean (SD))	0.37 (0.32)
Racial and ethnic minority status (mean (SD))	0.66 (0.22)
Housing type and transportation (mean (SD))	0.75 (0.21)

**Fig. 1:**
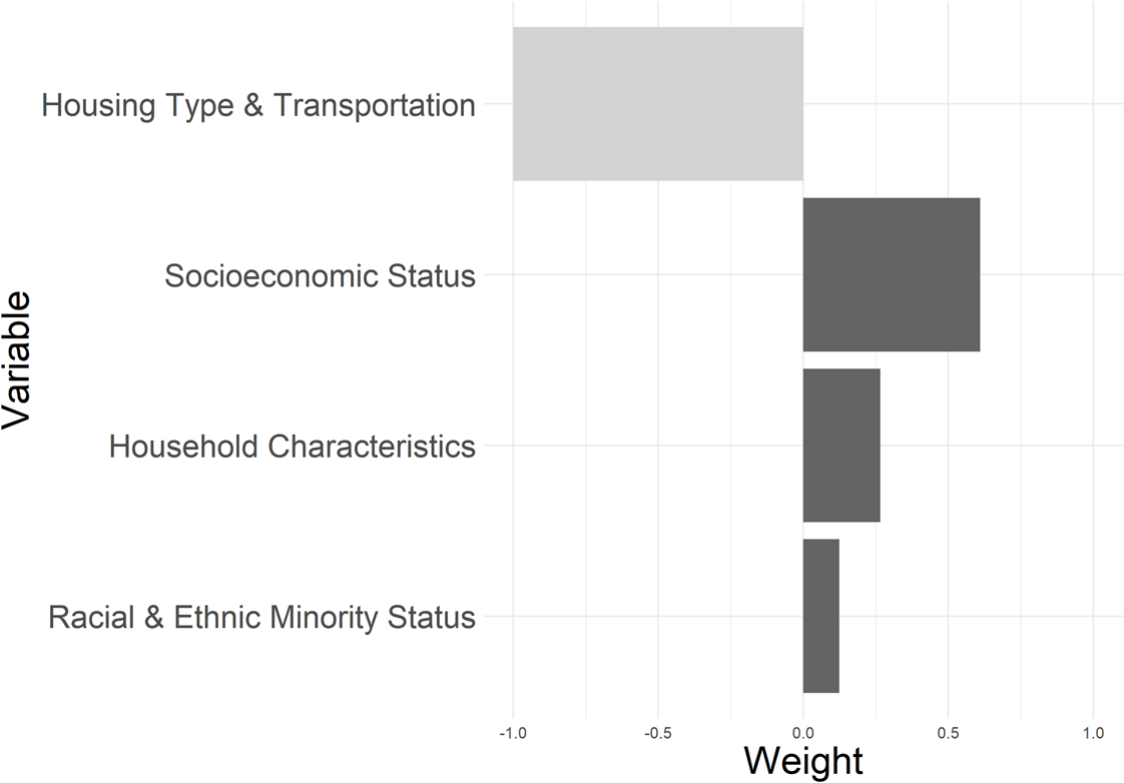
Weights representing the proportion of the positive and negative partial effect in a quantile g-computation model assessing the associations between the four SVI themes and PhenoAgeAccel. Darker shading indicates a stronger partial effect. Decile increase in all exposure was associated with an increase of 0.23 (95% CI 0.21, 0.25) in PhenoAgeAccel

In our qGcomp analysis, a decile increase in the mixture of SVI dimensions was associated with an increase of 0.23 years (95% CI 0.21, 0.25) in PhenoAgeAccel. The socioeconomic status dimension was the main driver of the association, accounting for 61% of the weight, followed by household composition and racial and ethnic minority status. Housing type and transportation had an insubstantial protective role (Fig. 1).

**Fig. 2:**
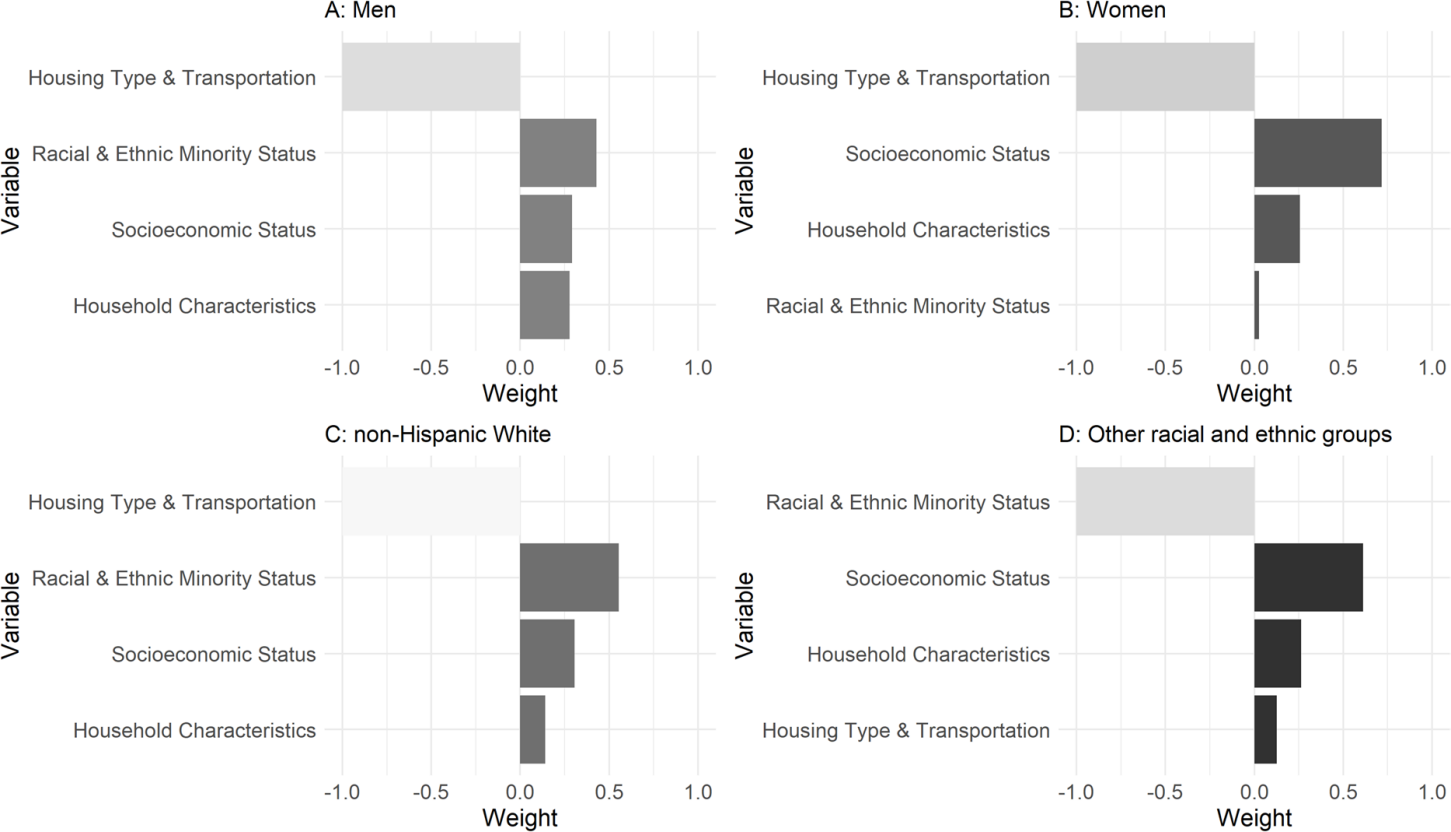
Weights representing the proportion of the positive and negative partial effect in stratified quantile g-computation models assessing the associations between the four SVI themes and PhenoAgeAccel. **A** For men, a decile increase in all exposures was associated with an increase of 0.13 (95% CI 0.10, 0.16) in PhenoAgeAccel. **B** For women, 0.27 (0.25, 0.29). **C** For non-Hispanic Whites, 0.12 (0.09, 0.15). **D** For other racial and ethnic groups, 0.34 (0.32, 0.36). Darker shading indicates a stronger association in the direction

Interaction models revealed an effect modification in the association between the SVI mixture and PhenoAgeAccel by sex. For females, a decile increase in the mixture of SVI dimensions was associated with an increase of 0.27 years (95% CI 0.25, 0.29), mainly driven by socioeconomic status (71%). For males, a decile increase was associated with an increase of 0.13 years (0.10, 0.16), mainly driven by racial and ethnic minority status (43%), socioeconomic status (29%), and household characteristics (27%). Interaction models also revealed an effect modification by race and ethnicity when comparing non-Hispanic White to the rest of the participants. For non-Hispanic Whites, a decile increase was associated with an increase of 0.12 years (0.09, 0.15), driven by racial and ethnic minority status (55%). For the other racial and ethnic groups, a decile increase was associated with an increase of 0.34 years (0.32, 0.36), driven by socioeconomic status (61%) (Fig. 2).

The characteristics of the person-years in the imputed analytical dataset did not differ from the population characteristics among the person-years with complete lab information (Table S3). Results of the sensitivity analysis limiting the sample to person-years with complete information (5853 participants and 8670 person-years) were very similar to the estimates observed in the imputed analytical dataset (Table S4). Lastly, cluster-bootstrapped models showed no evidence of substantial clustering among participants’ repeated measures, with very similar estimates and precision in the model with participant-based (0.23 years, 95% CI 0.19, 0.27) and census-tract-based bootstrapping (0.23 years, 95% CI 0.19, 0.26).

## Discussion

Our findings show that higher neighborhood-level social vulnerability is associated with an increase in PhenoAgeAccel. In other words, the social vulnerability of the participants’ neighborhood of residence accelerated their biological aging, therefore increasing their morbidity and mortality risk. The socioeconomic status theme is the primary driver of the association, while housing type and transportation had an insubstantial role. Interaction models revealed stronger associations among women and among racial and ethnic minorities.

Our results contribute to the growing body of evidence linking social vulnerability to diverse health outcomes, particularly aging outcomes. However, our study is the first to investigate the associations between social vulnerability and biological aging. In a cross-sectional US-wide analysis, county-level SVI was associated with an increase in cardiovascular disease-related mortality rate [37]. A Medicare-based analysis found higher risks of diverse postoperative surgical outcomes associated with SVI [38]. In a Canada-based cohort, self-reported social vulnerability has been associated with increased risk of mortality [39] and cognitive decline [40] as well as a decline in cognitive function [41] in older adults. In a European-based cohort, self-reported social vulnerability was associated with an increased risk of frailty [42].

Utilizing the overall SVI score—or any index or score that combines different aspects of social vulnerability—allows researchers to navigate the complex correlation structure of social, economic, and demographic variables, thus avoiding issues of collinearity and interpretation challenges [43]. However, the overall SVI score might oversimplify the underlying factors linking social vulnerability and biological aging [44]. Our mixture approach enables us to evaluate the effects of the different themes simultaneously, as they occur in real life. Doing so, we obtain a closer measure of the role effects of each theme, informing on how to best target policy to reduce the overall negative effect [45].

Our results highlighted neighborhood socioeconomic status as the main driver of the association, followed by household characteristics, and finally, racial and ethnic minority status. Housing type and transportation did not have a substantial role. The socioeconomic status domain was previously associated with increased cognitive decline [46] and function [47], self-reported health, and mortality among older adults [48]. Household structure has been previously linked to elderly mortality [49] and informal care [50]. Additionally, neighborhood racial and ethnic minority status has been associated with cognitive decline [51]. Moreover, in a cross-sectional US-wide study among the general population, SVI’s socioeconomic status and racial and ethnic minority status had the strongest associations with the presence of personal-level social determinants of health, including issues with social connections, health literacy, financial resource, transportation, food security, safety, and housing stability [52].

Neighborhood effects can vary from person to person based on the interaction between neighborhood conditions and personal attributes and behaviors [53]. We found females to be more susceptible to social vulnerability-related accelerated aging compared to males. Moreover, the weight of the themes was substantially different, with socioeconomic status driving the association for females and it being split between socioeconomic status, household characteristics, as well as racial and ethnic minority status in men. Similar to our study, a 158-participant study in Detroit found stronger associations between neighborhood quality and PhenoAgeAccel in females than in males, and a study in the UK also found stronger associations between neighborhood quality and self-reported health for women than for men [54]. Comparable results were obtained when stratifying by race and ethnicity. Social vulnerability was associated with a smaller PhenoAgeAccel increase in non-Hispanic White than in the other racial and ethnic groups. Similarly, an analysis using the Health and Retirement Study cohort reported stronger associations between neighborhood characteristics and telomere length in Black individuals than in White individuals [55]. Moreover, the association in non-Hispanic Whites is led by racial and ethnic minority status theme, but socioeconomic status leads in the other group.

The observed differences in the effects of social vulnerability between women and men, as well as between racial and ethnic minorities and non-Hispanic Whites, can be attributed to distinct mechanisms [56] involving perception—e.g., women might perceive and area as less safe than men [57], and interaction—e.g., due to continued exposure to stressors, racial and ethnic minorities might be better equipped to counteract them [57, 58]. Black [59, 60] and Hispanic [61, 62] individuals often experience worse aging outcomes compared to non-Hispanic Whites. In summary, individual-level characteristics can increase vulnerability while influencing the detrimental effects of neighborhood-level exposures, potentially combining into detrimental effects stronger than their separate ones.

This study is not without limitations. The formula for PhenoAge includes blood biomarkers that are not routinely drawn in the MSHS, potentially introducing selection bias. We have minimized this bias by imputing C-reactive protein and lymphocytes when those are the only two biomarkers missing. Moreover, the analysis being based on EHR data could reduce the sample’s representativeness. However, we include all available biomarkers—both inpatient and outpatient—and we have limited our sample to 65 years or older, a subpopulation who are more likely to visit the health system for regular check-ups. Our results regarding housing type and transportation might be linked to transportation and housing features specific to New York City. Therefore, our results might not be generalizable to other community types (i.e., rural) where transportation and resource access are more limited. However, these distinct transportation and housing features are common among most US metropolises. Due to epigenetic data being unavailable in our EHR-based cohort, we have focused on PhenoAgeAccel and did not include other validated biological age clocks. The benefit of our approach is the use of an equation that does not require specialized assays and is therefore transferable and easily replicable in different settings and geographic areas. Lastly, race and ethnicity were operationalized as binary variables in the interaction analysis because qGcomp limits the ability to include multiple categorical variables for interaction effects.

## Conclusions

Our study shows that higher social vulnerability is associated with an increase in biological age measured as phenotypic age acceleration. We observed that the association is led by socioeconomic status, followed by household characteristics and racial and ethnic minority status. At the same time, housing type and transportation seem to have an irrelevant role. These observed associations were especially pronounced among women and racial and ethnic minority groups. Our findings suggest that the social environment, in addition to personal-level stressors, plays an important role in healthy aging, pinpoint which facets of the social environment are the more relevant, and that vulnerability is differential among population groups.

Social environments are often represented by a single metric or index that fails to capture the complexity of a city’s social, economic, and demographic landscape. This makes the design and implementation of policies more difficult, especially when the specific needs of the most vulnerable populations are not considered and those needs are population-specific.

Lastly, our results shed light on the intersection between neighborhood-level social stressors and personal factors. For example, we found that living in a minority-dominated neighborhood is not a relevant factor in social environment-related biological age increases among racial and ethnic minority groups. This finding is crucial for targeting aging disparities with precision. As suggested by the geroscience hypothesis, focusing on biological aging as a modifiable process, therefore targetable with policy and action, offers an opportunity to delay morbidity and mortality. Addressing risk factors within the social environment may hold the key to shifting from treatment to prevention.

##  Supplementary Information

Below is the link to the electronic supplementary material.

Supplementary file1 (DOCX 228 KB)

## Data Availability

Social Vunerability Index data is openly available in the ATSDR webpage at https://www.atsdr.cdc.gov/placehealth/php/svi/index.html. The participants of this study did not give written consent for their data to be shared publicly, so due to the sensitive nature of the research supporting health data is not available.
