# Supplementary material for: Social Vulnerability and Biological Aging in New York City: An Electronic Health Records-Based Study
Source: J Urban Health. 2025 Jan 14;102(2):240–9. doi: 10.1007/s11524-024-00948-7 (PMC12031684; doi:10.1007/s11524-024-00948-7)

**Supplementary Material:**

**Table S1:** The current CDC-SVI incorporates 16 variables from the 5-year American Community Survey (ACS). These variables are categorized into four themes, each representing a key aspect of social vulnerability, and are then aggregated into a single comprehensive measure of overall social vulnerability.

| Theme | Variable |
| --- | --- |
| Socioeconomic Status | Below 150% Poverty |
|  | Unemployed |
|  | Housing Cost Burden |
|  | No High School Diploma |
|  | No Health Insurance |
| Household Characteristics | Aged 65 & Older |
|  | Aged 17 & Younger |
|  | Civilian with a Disability |
|  | Single-Parent Households |
|  | English Language Proficiency |
| Racial & Ethnic Minority Status | Hispanic or Latino (of any race) |
|  | Black and African American, Not Hispanic or Latino |
|  | American Indian and Alaska Native, Not Hispanic or Latino |
|  | Asian, Not Hispanic or Latino |
|  | Native Hawaiian and Other Pacific Islander, Not Hispanic or Latino |
|  | Two or More Races, Not Hispanic or Latino |
|  | Other Races, Not Hispanic or Latino |
| Housing Type & Transportation | Multi-Unit Structures |
|  | Mobile Homes |
|  | Crowding |
|  | No Vehicle |
|  | Group Quarters |

| **Table S2:** Descriptive statistics at baseline for the study population stratified by sex as well as race and ethnicity at baseline. Comparisons were performed using chi square test for categorical variables and ANOVA for continuous ones. | | | | | | |
| --- | --- | --- | --- | --- | --- | --- |
| **Variable** | **Female** | **Male** | **p-value** | **Non-Hipanic White** | **Other race and ethnicity** | **p-value** |
| n | 21044 | 10869 |  | 17949 | 13964 |  |
| Sex = Male (%) | 0 ( 0.0) | 10869 (100.0) | <0.001 | 5406 (30.1) | 5463 ( 39.1) | <0.001 |
| Age (mean (SD)) | 70.42 (8.64) | 69.81 (8.16) | <0.001 | 69.92 (8.50) | 70.59 (8.46) | <0.001 |
| Race and Ethnicity Combined (%) |  |  | <0.001 |  |  | <0.001 |
| *American Indian or Alaska Native* | 28 ( 0.1) | 7 ( 0.1) |  | 35 ( 0.2) | 0 ( 0.0) |  |
| *Asian* | 908 ( 4.3) | 586 ( 5.4) |  | 1494 ( 8.3) | 0 ( 0.0) |  |
| *Black or African-American* | 3787 (18.0) | 1318 ( 12.1) |  | 5105 (28.4) | 0 ( 0.0) |  |
| *Hispanic* | 5806 (27.6) | 2435 ( 22.4) |  | 8241 (45.9) | 0 ( 0.0) |  |
| *Native Hawaiian or Pacific Islander* | 13 ( 0.1) | 4 ( 0.0) |  | 17 ( 0.1) | 0 ( 0.0) |  |
| *Other* | 2001 ( 9.5) | 1056 ( 9.7) |  | 3057 (17.0) | 0 ( 0.0) |  |
| *White* | 8501 (40.4) | 5463 ( 50.3) |  | 0 ( 0.0) | 13964 (100.0) |  |
| Insurance Type (%) |  |  | <0.001 |  |  | <0.001 |
| *Medicaid* | 743 ( 3.5) | 381 ( 3.5) |  | 950 ( 5.3) | 174 ( 1.2) |  |
| *Medicare* | 15182 (72.1) | 7444 ( 68.5) |  | 12398 (69.1) | 10228 ( 73.2) |  |
| *Other* | 905 ( 4.3) | 530 ( 4.9) |  | 737 ( 4.1) | 698 ( 5.0) |  |
| *Private insurance* | 4023 (19.1) | 2395 ( 22.0) |  | 3690 (20.6) | 2728 ( 19.5) |  |
| *Self-pay* | 191 ( 0.9) | 119 ( 1.1) |  | 174 ( 1.0) | 136 ( 1.0) |  |
| Overall SVI (mean (SD)) | 0.59 (0.28) | 0.55 (0.27) | <0.001 | 0.71 (0.25) | 0.41 (0.23) | <0.001 |
| Socioeconomic status (mean (SD)) | 0.51 (0.31) | 0.47 (0.30) | <0.001 | 0.64 (0.28) | 0.31 (0.25) | <0.001 |
| Household Characteristics (mean (SD)) | 0.39 (0.32) | 0.34 (0.30) | <0.001 | 0.48 (0.33) | 0.24 (0.24) | <0.001 |
| Racial and Ethnic Minority Status (mean (SD)) | 0.66 (0.22) | 0.64 (0.21) | <0.001 | 0.76 (0.18) | 0.53 (0.19) | <0.001 |
| Housing Type and Transportation (mean (SD)) | 0.75 (0.21) | 0.74 (0.22) | <0.001 | 0.78 (0.20) | 0.71 (0.22) | <0.001 |

| **Table 1:** Descriptive statistics at baseline for the overall study population and non-imputed subsample at baseline. | | |
| --- | --- | --- |
| **Variable** | **Overall** | **Subsample without imputations** |
| n | 31913 | 5853 |
| Sex = Male (%) | 10869 (34.1) | 1883 (32.2) |
| Age (mean (SD)) | 70.21 (8.49) | 71.86 (8.43) |
| Race and Ethnicity Combined (%) |  |  |
| *American Indian or Alaska Native* | 35 (0.1) | 7 (0.1) |
| *Asian* | 1494 (4.7) | 249 (4.3) |
| *Black or African-American* | 5105 (16.0) | 975 (16.7) |
| *Hispanic* | 8241 (25.8) | 1500 (25.6) |
| *Native Hawaiian or Pacific Islander* | 17 (0.1) | 4 (0.1) |
| *Other* | 3057 ( 9.6) | 513 (8.8) |
| *White* | 13964 (43.8) | 2605 (44.5) |
| Insurance Type (%) |  |  |
| *Medicaid* | 1124 (3.5) | 168 (2.9) |
| *Medicare* | 22626 (70.9) | 4315 (73.7) |
| *Other* | 1435 (4.5) | 252 ( 4.3) |
| *Private insurance* | 6418 (20.1) | 1068 (18.2) |
| *Self-pay* | 310 (1.0) | 50 (0.9) |
| Overall SVI (mean (SD)) | 0.58 (0.28) | 0.58 (0.28) |
| Socioeconomic status (mean (SD)) | 0.50 (0.31) | 0.50 (0.31) |
| Household Characteristics (mean (SD)) | 0.37 (0.32) | 0.39 (0.32) |
| Racial and Ethnic Minority Status (mean (SD)) | 0.66 (0.22) | 0.66 (0.21) |
| Housing Type and Transportation (mean (SD)) | 0.75 (0.21) | 0.74 (0.21) |

| **Table S4:** Estimates from a quantile g-computation model assessing the associations between SVI and PhenoAgeAccel. Estimate for the complete sample and a subsample without imputations as well as overall and interaction models. Estimates represent the increase associated with a decile increase in SVI. | | |
| --- | --- | --- |
| **Model** | **Complete sample** | **Subsample without imputations** |
| Overall | 0.23 (0.21, 0.25) | 0.26 (0.19, 0.33) |
| Interaction – Females | 0.27 (0.25, 0.29) | 0.30 (0.22, 0.38) |
| Interaction – Males | 0.13 (0.10, 0.16) | 0.15 (0.03, 0.28) |
| Interaction – non-Hispanic Whites | 0.12 (0.09, 0.15) | 0.14 (0.015, 0.27) |
| Interaction – Other race and ethnicity groups | 0.34 (0.32, 0.36) | 0.39 (0.31, 0.48) |

**Figure S1:** Correlation structure amongst each SVI theme at baseline.


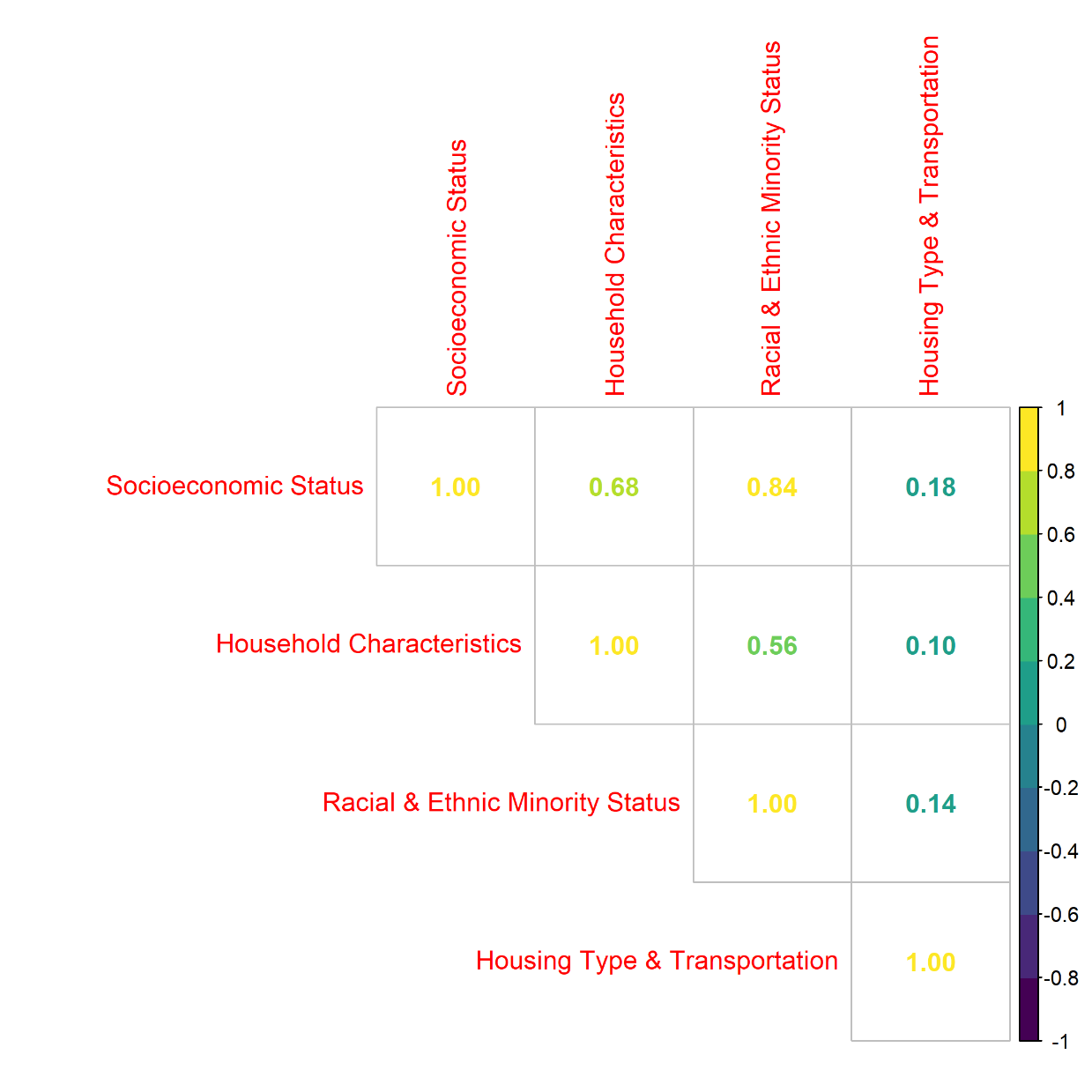

Supplement: Supplementary file 1 — Supplementary file1 (DOCX 228 KB) [file 11524_2024_948_MOESM1_ESM.docx]
